# Scarlatina Anginosa Maligna

**Published:** 1810-12

**Authors:** William Goodwin

**Affiliations:** Surgeon. Earlsoham, Framlingham, Suffolk


*To the Editors of the Medical and Physical Journal,*

---

*Scarlatina Anginosa Maligna.*

GENTLEMEN,

SCARLATINA Anginosa Maligna having made its appearance in its very worst forms, in a large district round Debenham, in Suffolk; I take the liberty of sending you the particulars attending several fatal cases, with the general plan, and medicines employed.

The first case appeared at Debenham in February last, commencing with a cold shivering fit; enlarged and ulcerated tonsils, surrounded with an erysipelatous purpley inflammation attended with grey coloured spots; in a day or two, the scarlet eruption appeared about the neck, face, and bosom, and afterwards extended to the extremities, and generally accompanied with stiffness and tumefaction of the wrists, fingers, ancles, and toes. Pain of the head and back, and very distressing heat, were the most usual symptoms complained of, together with great prostration of strength, and faintness, want of sleep, and a low tremulous pulse, from 110 to 140 in a minute.

On the 15th of April last, I was called to Mr. Pooley, a stout fat man of about 34, who was well and about his business on Friday, was taken in the evening with a rigor and sore throat, which, on examining was found with greenish spots, inflamed tonsils, with ill-conditioned ulcerations.

His skin was red, spirits and strength fallen, pulse low, and 115. Fumigations with vinegar, gargles with myrrh, cinchona and muriatic acid; sponging the surface with vinegar and water, were immediately and actively applied; an emetic and a calomel purge were immediately given, together with strong decoction of cinchona, serpentaria, aromatics, muriatic acid, and oxygenated muriatic acid. The danger being imminent, the pulse low, and the patient sinking, wine, and occasionally brandy, were given. He had every advantage from attentive nursing, a large well aired and ventilated room; but sunk under the disease on the 4th day, and in a few hours the strongest marks of putridity, and indeed rottenness, attended the dead body.

Mr. K———, a respectable farmer, was well at a friend's house, on Monday, the 30th of August; on that day, he had a cold chill and soreness and stiffness of his throat; on Tuesday was worse; on Wednesday complained of head-ache, pain of his back, great loss of strength, scarlet eruption, pulse low, low, tremulous, and 120. Calomel and an emetic were first given, and then antisceptics, acids, fumigations, &c. &c. as in the first case, were boldly tried, but without effect; we having the mortification to see him die on the 4th day, in the 31st year of his age.

Mr. Garnham, 30 years of age, was at work on his farm, on Tuesday the 2d of August, was chilly on Wednesday; his throat being stiff and sore, he rode three miles for an emetic, was worse on Thursday, throat swelled, and much ulcerated, tongue and fauces very foul, distressing head-ache, pulse 140, urine high-coloured, and countenance yellow, which more or less was the case with most of those who had the disease in its worst form.

He died on the 5th day, and the body almost immediately became putrid and rotten.

I forbear reciting more of these melancholy cases, of which we have had but too many; some, even children, have died in 48 hours from the first attack. All the Surgeons for ten miles round, have had to attend Scarlatina Maligna in a variety of cases, in all ages from infancy to 50 or 60 years, and all have had their share of losing their patients.

The disease appears in various forms, from the slightest affection, with little or no sore throat or eruption, to the very worst and malignant type, which seems to baffle all attempts at stemming its violence. Mr. Torrence's cases of Scarlatina in your 185th No. appear of a milder kind, and much less formidable than many of those we have encountered, or cold effusion could scarcely have stemmed the course of the disease; and indeed, however valuable Dr. Curry's plan may be in Scarlatina, or other fevers, unless it was *infallible*, country practitioners dare not put it in use to its *fullest extent*; for if two or three lives of any consequence were lost after trying it, all the relatives, nurses, and prejudiced persons would exclaim against the Physician and Apothecary as murderers and barbarous practitioners.

The best writers on the putrid malignant sore throat, from Sydenham, Pringle, &c. down to Dr. Hooper, advise the tonic, antiseptic, aromatic, ascessent, and cordial plan, which have been tried most vigorously in a number of cases, not forgetting blisters over the throat. For children, and those that could not use the gargle, or admit of syringing, a linctus was ordered with mel. borac. alum. rup. pip. cayen. and acid. muriat. The state of the bowels was carefully attended to in all, and in some cases, injections of decoct. cinchon. vin. rubr. R. opii and acet. were administered by the bowels.

Although the mortality has been greater than I can recollect lect in the course of the last 40 years, we have the pleasure to record the escape of by very far the greater number of the afflicted with Scarlatina Anginosa. The best writers on this cruel malignant fever, and the most able practitioners of my acquaintance, many of whom I have met, all concur in advising the ionic and antiseptic plan, which has been actively employed, modifying it according to the circumstances of the different patients, and degrees of malignancy attending; but I am truly sorry to say, without the wished for success in too many, or indeed, without any marked benefit at all in several.

This disease has in some instances afflicted whole families, with various degress of malignancy, from a slight sore throat to the highly putrid, causing death. In others, a part only have the disease at all. Many families are now suffering in different spots around this country, that appear to have had no communication with the afflicted. In one family, a child fell ill with Scarlatina, in its mildest form; the father, mother, and another child, fell down on the seventh day from the first: the father died, the others recovered. Since writing the above, I was called to a Mr. Rose, whose wife died of Scarlat. Ang. Malig. two days before; her body was so highly putrid, that the house was hardly bearable. Rose's symptoms were truly alarming from putrid ulcers in the throat, scarlet eruption, low quick pulse, and delirium, with two children in the same dangerous state, but those all recovered after the most severe struggle. Your medical friends will do the profession and the public the greatest favour, if they will point out in your next Journal, a more proper and successful mode of treating the Scarlatina Anginosa, under the highly malignant and rapidly destructive form it has assumed in this spot. Indeed my troubling you with this hasty sketch, is chiefly for the purpose of soliciting advice and instruction from the more learned and able, than,

Gentlemen,

Your obedient Servant, &c.

WILLIAM GOODWIN, Surgeon.

*Earlsoham, Framlingham, Suffolk.*

P. S.—*Oct.* 22, 1810.—This malignant fever still rages around us; many dying in 48 hours after being attacked.

